# Muscular Activity Modulation During Post-operative Walking With Hybrid Assistive Limb (HAL) in a Patient With Thoracic Myelopathy Due to Ossification of Posterior Longitudinal Ligament: A Case Report

**DOI:** 10.3389/fneur.2020.00102

**Published:** 2020-03-31

**Authors:** Hideki Kadone, Shigeki Kubota, Tetsuya Abe, Hiroshi Noguchi, Kousei Miura, Masao Koda, Yukiyo Shimizu, Yasushi Hada, Yoshiyuki Sankai, Kenji Suzuki, Masashi Yamazaki

**Affiliations:** ^1^Center for Innovative Medicine and Engineering, University of Tsukuba Hospital, Tsukuba, Japan; ^2^Center for Cybernics Research, University of Tsukuba, Tsukuba, Japan; ^3^Department of Orthopaedic Surgery, Faculty of Medicine, University of Tsukuba, Tsukuba, Japan; ^4^Department of Rehabilitation Medicine, University of Tsukuba Hospital, Tsukuba, Japan

**Keywords:** myelopathy, gait recovery, Hybrid Assistive Limb (HAL), ossification of posterior longitudinal ligament (OPLL), muscle activity analysis, synergy analysis, muscle network analysis, exoskeleton robot

## Abstract

Disorders of the central nervous system sometimes cause severe sensory motor paralysis accompanied by gait impairment. Recently, there are several reports on the effectiveness of robot-assisted gait training for patients experiencing these issues. The purpose of this case report was to assess the neuromechanical effect of a wearable robot suit HAL (Hybrid Assistive Limb) during post-operative gait training in a patient with gait impairment due to compressive myelopathy caused by ossification of the posterior longitudinal ligament (OPLL). For this purpose, we compared lower limb muscular activities while the patient was walking with and without the robot through a course of treatment sessions by (i) gait phase-dependent muscle usage analysis, (ii) muscle synergy analysis, and (iii) muscle network analysis. The results show (i) enhanced activity of the extensor muscles for weight-bearing in the initial sessions by using HAL and reduced knee extensor and increased hip extensor activations for achieving larger steps and faster gait in the later sessions; (ii) involvement of a greater number of synergies during walking with HAL than without HAL; and (iii) modulated muscle network property during walking with HAL remaining until the next HAL session. The patient's gait was improved after completing HAL sessions, acquiring close to normal joint profile with greater range of joint movement, faster walking speed, and larger step length. We discuss that the muscular activity modulation during walking with HAL suggests altered control of the muscles by the central nervous system during post-operative walking. Activity-dependent sensorimotor augmentation by HAL is discussed in the context of recovery of gait control by the central nervous system. The relationship between the altered control and the achieved gait recovery requires further investigation.

## Introduction

Disorders of the central nervous system sometimes cause severe sensory motor paralysis accompanied by gait impairment. There are several recent reports on gait improvement after clinical intervention using wearable exoskeleton-type robots in patients with gait disturbance after central nervous system disorders; for example, ReWalk (1), Indego (2), Ekso (3), Lopes (4), Lokomat (5, 6), MindWalker (7), and HAL (8).

Robot suit HAL (Hybrid Assistive Limb; Cyberdyne, Tsukuba, Japan) assists motion of the bilateral hip and knee joints during walking in accordance with voluntary motion intension of the user (9). It actuates the electric motors embedded in the hips and knees of its exoskeleton in real time, amplifying bioelectric activation of the relevant muscles which are detected using surface electrodes attached on the hip and knee muscles. Previous studies using HAL for myelopathy (10–12), spinal cord injury (13–17), and post-surgery rehabilitation after total knee arthroplasty (18) reported improvement of walking ability after HAL training.

Ossification of the posterior longitudinal ligament (OPLL) is characterized by heterotopic ossification of the ligament usually at the cervical and thoracic spine (19, 20). Stenotic reduction of space for the spinal cord within the spinal canal due to the ossification induces spinal cord compression, resulting in severe myelopathy. The myelopathy is degenerative, categorized similarly to those caused by other reasons, including ossification of the ligamentum flavum, degenerative disc disease, and spondylotic myelopathy (21). Surgical treatment for spinal cord decompression is recommended when symptomatic neurological deterioration is observed, including gait disturbance, bladder disorder, and a myelopathic hand (22–24); otherwise, the risk of cervical spinal cord injury is suggested (25, 26). After decompression surgery, patients are prescribed with a rehabilitation program.

Analysis of myelopathic gait can be found for patients with cervical myelopathy due to cervical spondylosis and/or OPLL who were able to autonomously ambulate before and after decompression surgery (27). Compared with cervical myelopathy, neurological deterioration in thoracic myelopathy is rather concentrated in the lower limbs: lower limb numbness and weakness and an unsteady gait (28). Post-operative motor paralysis was observed in more than 30% of thoracic patients who underwent surgery for OPLL, more than 20% of whom underwent additional surgery to achieve recovery (29). Post-operative paralysis of thoracic OPLL patients tends to be severe, in accordance with the duration and severity of compression before surgery, even when enough decompression is obtained by surgery (30, 31). While the existing studies on gait of myelopathy patients deal with patients who could ambulate autonomously before and after surgery (27), here, we consider a case who had difficulty walking before and after surgery for thoracic OPLL.

The spinal cord has been considered to be housing the spinal locomotor network which coordinates the rhythmic limb motion during locomotion, based on evidences from non-human species and partly from humans (32, 33). For the recovery of spinal locomotor function after injury, the importance of sensory input of movement based on activity is suggested (34, 35). As well, the importance of task-specific active training is suggested to enhance plasticity of the supraspinal neural networks to adapt to the injured spinal cord in the generation of a closer to normal gait (36). Since HAL assists activity-based joint motion during locomotion and, hence, assists in providing coherent sensory input to the spinal cord through assistance of actual performance of the motion intended by the neural system, we think that HAL can provide an effective method for the recovery of gait after spinal cord disorder. Actually, gait improvement after training using HAL has been reported for patients after spinal cord injury (references cited above), among which, Shimizu et al. (17) reported reactivation in some of the paralyzed muscles of chronic spinal cord injury patients during walking with HAL. Compressive myelopathy and spinal cord injury are both spinal cord disorders leading to disturbance or impairment of gait, the difference being that the former is caused by chronic degeneration while the latter is caused by acute injury. Sensory input to the spinal cord achieved by HAL during walking is expected to be effective also for gait improvement of patients with severe thoracic myelopathy, bringing changes to the way the nervous system controls muscles during walking.

In previous studies on the application of HAL for post-operative gait rehabilitation of OPLL patients with thoracic myelopathy (10–12, 37–39), gait improvement is reported by comparing the state of the patient before starting and after finishing the entire robot-assisted intervention. However, this comparison hinders examination of the effect of the robot during the training *per se*, as well as how it differs from training without the robot. In this study, the gait and muscle activity during walking using the robot were recorded and analyzed in a patient with gait impairment due to thoracic myelopathy caused by OPLL. The purpose of this study was to examine the immediate effect of the robot on gait control and to discuss how it varies through intervention sessions. As far as we know, this is the first study showing the muscular activity changes during walking using HAL in a patient who had post-operative gait impairment after myelopathy. In this study, we analyze and compare the lower limb muscle activities during walking with and without HAL in each session of post-surgery rehabilitation. The analysis is made of three parts: gait phase-dependent amplitude analysis, muscle synergy analysis, and muscle signal network analysis.

Muscle synergy analysis is based on the idea that coordinated control of multiple muscles by the central nervous system is structured with the combination of a comparatively smaller number of basic synergy patterns (40) to simplify the control strategy by reducing the number of controlled variables (41–43). The neural basis of muscle synergies is reported as housed in the spinal cord (44) and the formation of spatiotemporal synergies by the brain (45) based on evidence from non-human species. Muscle synergy analysis is used to assess the efficacy of rehabilitation (46) as a physiological marker in patients suffering from stroke or trauma (47), an assessment tool of motor coordination in patients with cerebral palsy (48), and as a tool to evaluate recovery of bilateral control in stroke patients (49, 50). While an altered muscle synergy of incomplete spinal cord injury patients during walking has been reported (36, 51), there are no reports so far concerning muscle synergies of myelopathy patients, as far as we searched.

Muscle network analysis (52) is a rather recently proposed method to investigate the structure of the signal network among muscles by analyzing the measured electromyography (EMG) in the frequency domain using methods of coherence analysis and complex graph analysis (52). It is based on the findings that the synchronous rhythm of neural firings coordinates the whole body signaling of the neural system, including the brain and reaching to the muscles (53, 54). Muscle network analysis and coherence analysis are utilized in several literatures to investigate the central nervous system's control of motion in a pathologic population (55, 56). This is the first study to apply muscle network analysis to gait of myelopathy patients.

## Case Presentation

### Patient

The case was a 64-year-old male patient (height, 165 cm; weight, 90 kg; body mass index, 33 kg/m^2^) who had severely paralyzed lower limbs. The case, having had spent 10 years without subjective perception of symptoms after obtaining a diagnosis of cervical ossification of the posterior longitudinal ligament (OPLL) and lumbar spinal stenosis (LSS), was sent to a nearby hospital by emergency transportation after having difficulty standing. Frequent falls were experienced, about a month prior to this event. At the hospital, paralysis of the lower limbs was recognized by manual muscle test (MMT) as 0, 1, and 2 on the right and 2 and 3 on the left, together with sensory paralysis on the trunk and the lower limbs. The case was then transferred to our hospital for consideration of surgical treatment.

**Figure 1:**
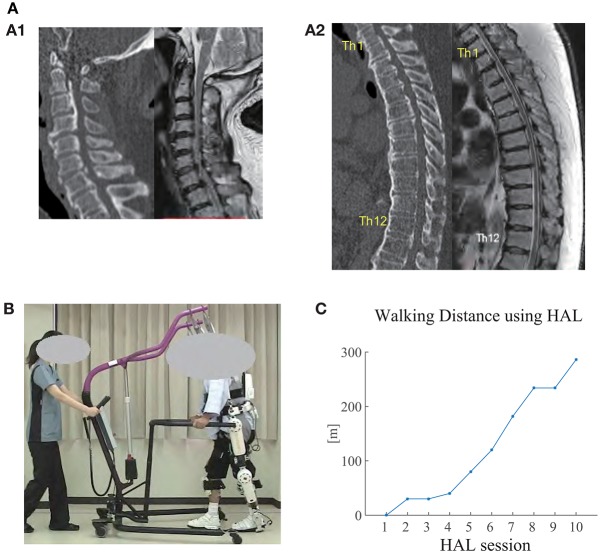
**(A)** CT (left) and MRI (right) images of the cervical spine (A1) and thoracic spine (A2) before surgery. Ossification of posterior longitudinal ligament (OPLL) was observed in the cervical and thoracic spine (from *C2* to *L1*), where the spinal cord was being compressed. Diffuse idiopathic skeletal hyperostosis (DISH) was also observed from *C4* to *L2*. Herniated disc was observed at Th8/9. **(B)** The patient walking overground using Hybrid Assistive Limb (HAL) in the seventh HAL session. An All-in-One Walking Trainer device was also used to provide weight support and for safety. An assistant pulled the device in accordance with the patient's gait. **(C)** Walking distance using HAL in each session. The distance gradually increased through the sessions.

At the moment of transfer, MMT scores deteriorated on the right lower limb (Table 1, Before Surgery). Voluntary movement of the limb was not possible lying on a bed. The left lower limb maintained the MMT scores and showed voluntary movement slightly; however, standing was not possible. Sensory disorder included hypesthesia on the trunk and the bilateral lower limbs, and pain and dysesthesia around the trunk and bilateral groin regions. CT and MRI showed compression on the spinal cord due to cervico-thoracic OPLL ranging from C2 to L1 (Figures 1A1,A2). On these examinations, thoracic myelopathy due to compression by the OPLL and instability at Th6/7, Th7/8, and Th8/9 were considered as the principal causes of the pathology. Surgical treatment was performed, including C3-T1 laminoplasty and Th2-12 posterior decompression and fusion, following which gradual improvement of the lower limb motor functions was observed. Posture training was started in a seated posture 9 post-operative days (POD), in a standing posture using a tilt table 10 POD, and then using parallel bars at 28 POD. Lower limb MMT scores improved, but then stayed flat for 20 days, which led to the prescription of HAL gait training at 43 POD (Table 1). The patient was in our hospital for 83 days before being transferred to another hospital for continuing treatments.

**Table 1:** Clinical assessment scores pre- and post-HAL.

		**Before surgery**	**23 POD**	**PRE (43 POD)**	**POST (72 POD)**
FIM-Motor		N/A	N/A	46	59
Barthel index		N/A	N/A	65	70
FAC		0	0	0	2
10–m walk test	Speed (m/min)	N/A	N/A	N/A	30.1
	Step length (m)	N/A	N/A	N/A	0.37
	Cadence (steps/min)	N/A	N/A	N/A	72.9
MMT (R/L)	Hip flexor	0/1	2/2	2/2	3/3
	Knee extensor	0/1	3/3	3/3	4/4
	Ankle dorsi-flexor	1/3	2/3	2/3	3/4
	Ankle plantar-flexor	0/2	3/3	3/3	3/3

*POD, postoperative days; FAC, functional ambulation category; MMT (R/L), manual muscle test (right/left); N/A, not applicable*.

### Hybrid Assistive Limb

A double-legged HAL was used in this study. HAL has an exoskeleton structure corresponding to the pelvis, bilateral thighs, shanks, and feet of the patient, weighing 14 kg in total. Bracings, hinged plates, and shoes are used to tighten the exoskeleton structure to the user by belts. It has electric motors at the bilateral hip and knee joints of the exoskeleton to assist sagittal motions of the hip and knee joints of the user. The electric motors are actuated by amplifying the bioelectric neuromuscular activation of the relevant muscles to support voluntary joint motions. In mechanical sense, the interaction of HAL and the user is based on the force that they apply to each other through the bracings, supporting plates, and shoes. Disposable surface electrodes (Vitrode L, Nihon Kohden, Tokyo, Japan) were attached to the HAL's cables and pasted bilaterally on the surface of the hip flexor (tensor fasciae latae), hip extensor (gluteus maximus), knee extensor (vastus lateralis), and knee flexor (biceps femoris) muscles of the patient. The motor torques were generated in real time in accordance with the user's muscle activity: T_hip = *G*_hip_flex*A_hip_flex-*G*_hip_ext*A_hip_ext and T_knee = *G*_knee_flex*A_knee_flex-*G*_knee_ext*A_knee_ext are the hip and knee joint torques, where *G*_^*^ are gain parameters, and *A*_^*^ are the filtered activation of the muscles, respectively. The motors' force is transmitted to the user's limbs through the exoskeletal structure, the bracings, and shoes to realize intended limb motions. The gain parameters were manually adjusted for the patient's comfort during walking in each session.

### HAL Sessions

Gait training using HAL (Figure 1B) was applied starting at 43 POD, followed by two or three sessions per week, 10 sessions in total, spanning a 28-day period. One session lasted about 1 h, including a 10-m walking test without using HAL (NoHAL), attachment of HAL, gait training using HAL (HAL), and detachment of HAL. HAL gait training included 20 min of overground walking activity at a comfortable pace on a 25-m oval course, with rest interval. All-in-One Walking Trainer (Ropox A/S, Naestved, Denmark) was used with a harness to provide weight-bearing support, in all walking (NoHAL and HAL) in all sessions. Weight support was manually adjusted in each session to provide minimum necessary weight support for the patient to keep walking in an appropriate posture. The All-in-One and HAL are completely separate and independent systems. There was no systematic interaction or shared control between them.

### Measurement

Pre–post-evaluations before the first HAL session (PRE) and after the last HAL session (POST) included clinical assessment of MMT of the major lower limb muscles, Functional Independence Measure—Motor General (FIM-M), Barthel Index (BI), functional ambulation category (FAC), and the 10-m walking test. During the 10-m walk test, gait kinematics was recorded using a motion capture system (Vicon MX with 16 T20s cameras, Vicon, Oxford, UK) sampling at 100 Hz. Sixteen autoreflective markers were placed on the anatomical landmarks according to a Plug-in Gait marker set: anterior superior iliac spine, posterior superior iliac spine, lower lateral 1/3 surface of the thigh, flexion–extension axis of the knee, lower lateral 1/3 surface of shank, lateral malleolus for the ankle, posterior peak of the calcaneus for the heel, and the second metatarsal bone of the toe.

In all HAL sessions, lower limb muscle activity was recorded during walking without HAL (NoHAL) and during walking with HAL (HAL) using a wireless surface EMG measurement system (Trigno Lab, Delsys, Natick, MA, USA) sampling at 2 kHz. In both NoHAL and HAL, the patient walked with the All-in-One walking device. EMG sensors were placed bilaterally on the gluteus maximus, vastus medialis, medial hamstrings, tibialis anterior, and medial gastrocnemius muscles after cleaning the skin over the muscle bellies with alcohol swabs. Foot marker movements were recorded at the same time in synchronization with EMG using the motion capture system for the purpose of gait phase detection.

### Data Processing

Marker positions of the motion capture data were converted to joint angles using the Plug-in Gait model of Vicon Nexus software (version 2.2.3). The rest of the processing was performed using custom scripts on MATLAB 8.4 R2014b (MathWorks, Natick, MA, USA). The flexion/extension angle of the hip and knee joints and the dorsi/plantar flexion angle of the ankle joint were extracted and divided into steps according to the height of the toe and heel markers. EMG data were first filtered with a band-pass filter (30–400 Hz), rectified and locally integrated using a 200-ms moving window to obtain an integrated EMG (iEMG) profile, which was then divided into steps according to the height of the toe and heel markers. The joint angle and iEMG profiles of each step were time-normalized to 101 points, with 0% representing a heel strike which initiates a cycle and 100% representing the subsequent heel strike which terminates the cycle. Data of multiple steps were averaged on the normalized time domain to obtain an averaged joint angle and iEMG profile of an averaged step. The averaged angle profiles were used to compare gait between PRE and POST, and the averaged iEMG profiles were used to compare muscle activity between NoHAL and HAL of each session. Gait was also compared between NoHAL and HAL by the ratio of total iEMG during the stance phase of the measured muscles.

Muscle synergy analysis was also used to compare the muscle activities between HAL and NoHAL. iEMG of the measured 10 muscles was decomposed into muscle synergy patterns and temporal coefficients by the non-negative matrix factorization (NNMF) method (57) against each of the possible number of synergies ranging from 1 to 10. Supposing that there are *m* muscles, *n* data samples, and *k* synergies, the factorization algorithm gives a decomposition of the muscle activity matrix *M* (*m* × *n*) into *M* = *SC* + *E*, where *S* is an *m* × *k* matrix containing *k* muscle synergies, *C* is a matrix containing temporal coefficients, and *E* is residual. For each of the assumed number of synergies, the fitting of the synergy patterns to the original iEMG patterns was evaluated in terms of variances accounted for (VAF) to estimate the number of synergy patterns underlying in the measured EMG data (58). In equation, $VAF=100\frac{{\left(\Sigma_{i}\Sigma_{j}X_{ij}Y_{ij}\right)}^{2}}{\left(\Sigma_{i}\Sigma_{j}{X_{ij}}^{2})(\Sigma_{i}\Sigma_{j}{Y_{ij}}^{2}\right)}$, where *X*_*ij*_ is the muscle activity and *Y*_*ij*_ is the reconstructed muscle activity of the *i*th muscle in the *j*th sample.

Muscle network analysis (52, 55) was also used to compare the muscle activities between HAL and NoHAL. Following the references, raw EMG data of each measured muscle was band-pass-filtered (20–70 Hz), resampled at 200 Hz, and rectified using Hilbert transform. The power spectral density (PSD) of each muscle was evaluated first to investigate the frequency range where the EMG signals have power. Welch's method was used here, with window length 1 s and overlap 0.75 s. Then, intermuscular coherence (IMCOH) was computed using Welch's method and the same window parameters for all pairs of the measured muscles. Coherence is commonly used to investigate coupling between neural activities (59, 60).

These data were then used to calculate the network measures clustering coefficient (CC) and global efficiency (GE) of a muscle network graph whose nodes are the measured muscles and whose arcs between the nodes are weighted by the IMCOH values. This gives a network representation of the measured muscles with their pairwise connectivity in the frequency domain. Supposing *w*_*ij*_(*f*) IMCOH of the *i*th and *j*th muscles after normalization in frequency domain, we calculated CC as the average of triplet multiplication of the connection weights 3*w*_*ij*_(*f*)*w*_*jk*_(*f*)*w*_*jk*_(*f*), sufficing *i* ≠ *j*, ≠ *k, k* ≠ *i*. GE was calculated as the average of the connection weights *w*_*ij*_(*f*), sufficing *i* ≠ *j*. CC and GE were, respectively, averaged through a lower (2–22 Hz) and a higher (22–44 Hz) frequency range. CC is known to provide an indication of the extent of functional segregation of a network, while GE indicates the extent of functional integration of a network (61). We then, in order to investigate the causal relationship of CC and GE between HAL and NoHAL, CC and GE during HAL were tested against, respectively, CC and GE during NoHAL in the next session using a linear regression.

## Results

### HAL Sessions

The patient completed 10 HAL sessions without any serious adverse events. The observed issues were redness on the skin due to attachment of the electrodes and minor scratches due to interference with belts and harnesses. All of these cutaneous issues disappeared promptly.

The patient did not manage to walk using HAL in the first session, in which stand-up training was provided in place. In the third session, gait data was not recorded because the patient reported perception of fatigue. Starting from the second session, the patient monotonically increased the walking distance using HAL (Figure 1C).

### Pre- Post-comparison

Clinical assessments (Table 1) showed improvements after HAL in the FIM-M score, BI, and FAC. The 10-m walking test was not available in PRE because he could not manage to complete 10 m even with weight support. The 10-m walking test in POST was completed using the handrail of the walking device with a practical walking speed, step length, and cadence. MMT scores improved after the surgery in most of the muscles and improved after HAL in some of the muscles.

**Figure 2:**
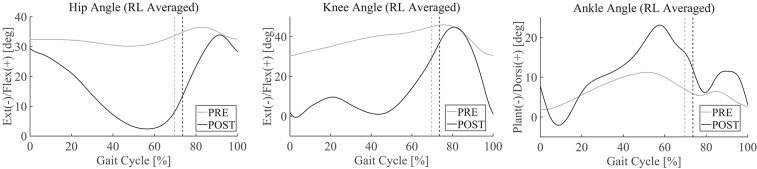
Joint angle profiles in the sagittal plane PRE [before starting the first Hybrid Assistive Limb (HAL) session] and POST (after the last HAL session). Joint angles from the right and left sides are separated into steps and averaged. 0% corresponds to a heel contact and 100% corresponds to the subsequent heel contact on the same side. *Vertical lines* indicate the moment of toe lift.

**Figure 3:**
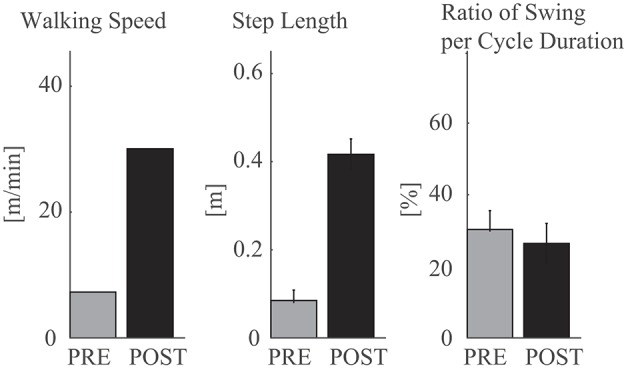
Gait parameters during walking in PRE and POST.

Pre–post-joint angle comparison demonstrated greater extension of the hip joint during stance and greater range of flexion motion during swing in POST than in PRE (Figure 2, left). In PRE, when the patient did not complete the 10-m walk, the joint angles and gait parameters were extracted from the several steps that he managed to perform. The knee joint showed greater extension during stance and greater range of flexion and extension during swing in POST than in PRE, and double knee action for shock absorption was observed in the earlier phase of stance in POST (Figure 2, middle). The ankle joint showed greater range of motion through the cycle, with greater planter flexion in the early phase of stance and greater dorsi-flexion at the end of stance and initial swing in POST than in PRE (Figure 2, right). Walking speed was faster and step length was greater in POST (Figure 3, left and middle). The swing-to-cycle duration ratio was comparable between PRE and POST (Figure 3, right).

### Gait Changes in HAL Sessions

**Figure 4:**
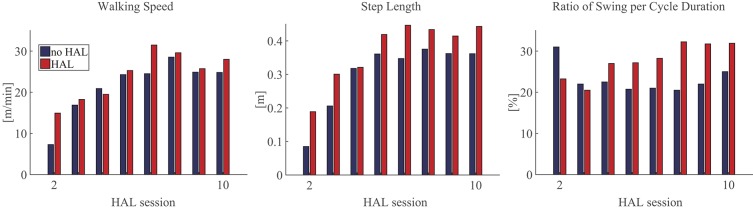
Gait parameters during walking with HAL (*HAL*) and without HAL (*NoHAL*) in each of the Hybrid Assistive Limb (HAL) sessions.

Comparing gait with HAL (HAL) and without HAL (NoHAL), the walking speed and step length were greater in HAL than NoHAL through the sessions (Figure 4, left and middle). The ratio of swing duration to cycle duration increased through the sessions in HAL and decreased in NoHAL; it was greater in HAL at the initial sessions and in NoHAL in the later sessions (Figure 4, right). Cadence was greater in NoHAL in most sessions (Supplementary Figure 1).

### Muscular Activity Changes in HAL Sessions

**Figure 5:**
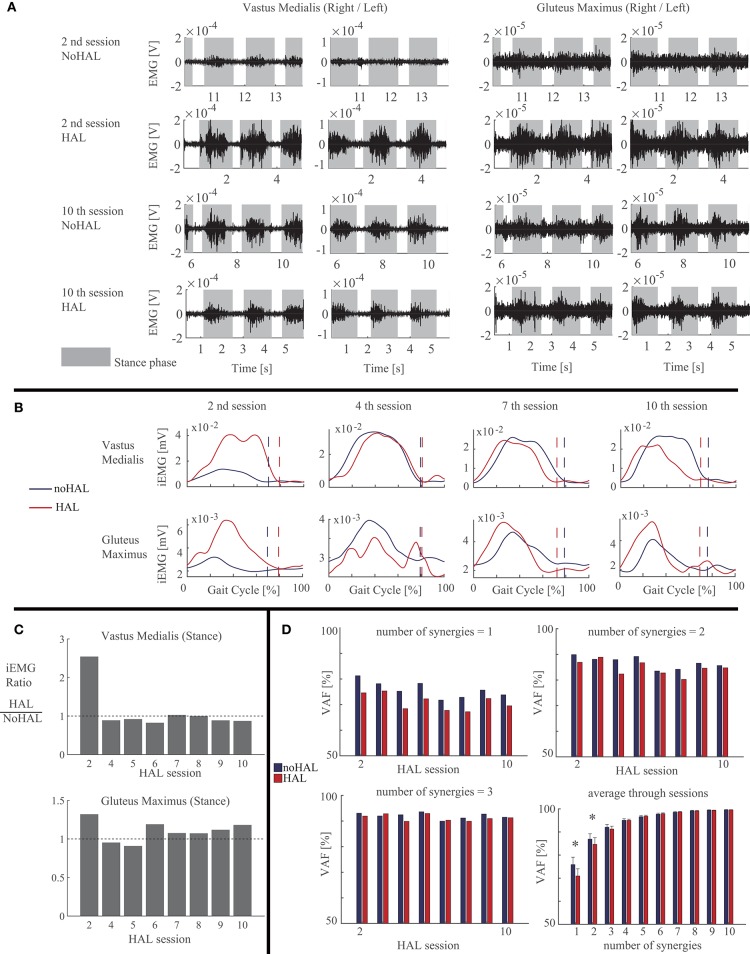
Activity of the quadriceps and gluteus maximus muscles compared during walking with HAL (*HAL*) and without HAL (*NoHAL*), in each of the Hybrid Assistive Limb (HAL) sessions. **(A)** Band-pass-filtered EMG data of the right and left sides in the second and 10th HAL sessions. The *gray shaded areas* indicate the stance phase. **(B)** Muscle activity profile through a gait cycle, averaged from multiple steps, in the second, fourth, seventh, and 10th HAL sessions. **(C)** Ratio of muscular activities during walking with HAL with respect to without HAL in each of the HAL sessions. **(D)** Variances accounted for (*VAF*) ratios representing the ratio of the measured muscle activities that can be reconstructed using the computed muscle synergies, for the cases of the number of muscles synergies being 1 (top left), 2 (top right), and 3 (bottom left). Bottom right shows the mean VAF averaged among the sessions for each case of the number of muscle synergies varying from 1 to 10 (*p < 0.05, by a paired *t*-test).

Activation of the vastus medialis (VM) and gluteus maximus (GM) during stance was greater in HAL than in NoHAL in the second session (Figures 5A,B), but not in the fourth session (Figure 5B). Activation of VM was smaller and GM was greater in HAL than in NoHAL during stance in the seventh and 10th sessions (Figures 5A,B). The ratio of activation of VM in HAL per NoHAL during stance (Figure 5C, left) showed that the activation was initially increased with HAL (second session) and then decreased in the later sessions, typically in the 10th session. As for GM (Figure 5C, right), the activation was initially increased with HAL (second session), then slightly decreased (fourth and fifth sessions), and then increased through the later sessions.

Muscle synergy analysis showed smaller VAF in HAL than in NoHAL in each of the sessions in cases of one and two synergy patterns (Figure 5D, top left and right, respectively). Averaging through the sessions showed a significantly smaller VAF in HAL than in NoHAL in cases of one and two synergies (*p* < 0.05; Figure 5D, right bottom). Since VAF represents the percentage of reconstruction using the restricted number of synergy patterns, the smaller VAF in HAL meant involvement of a greater number of synergy patterns in HAL than in NoHAL.

**Figure 6:**
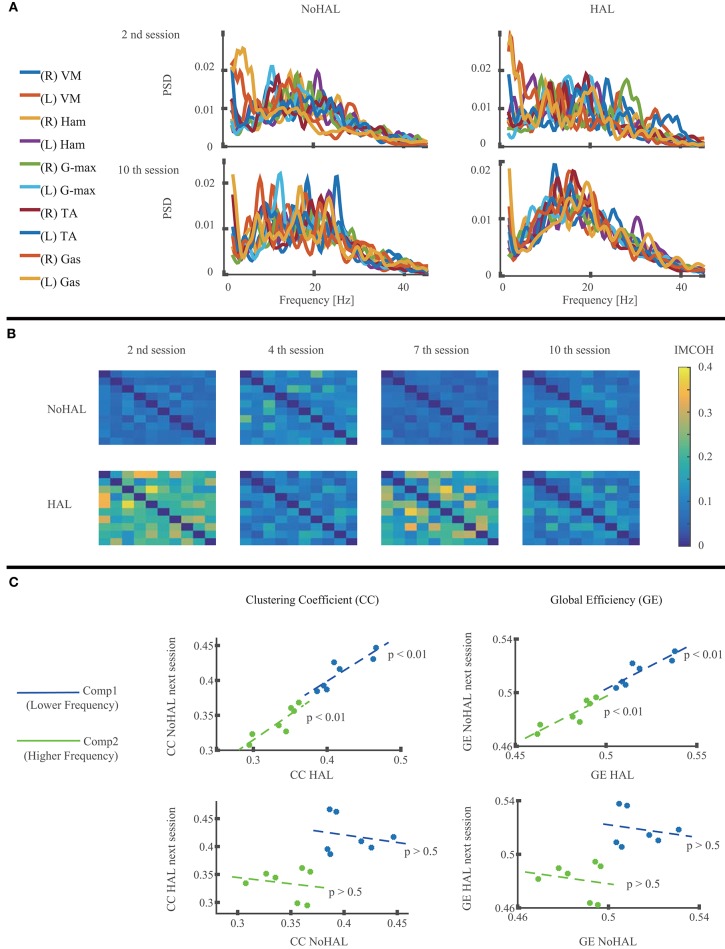
Muscle network analysis of EMG during walking without Hybrid Assistive Limb (HAL) and with HAL in each session. **(A)** Power spectral density (PSD) of the measured muscles in the second and 10th HAL sessions. **(B)** Intermuscular coherence (IMCOH) between the measured muscles in the second, fourth, seventh, and 10th HAL sessions, averaged through frequency. The order of the muscles is the same as in the muscle list in **(A)**, from top to bottom and from left to right. Color scale is shown on the right. **(C)** Linear regression of the clustering coefficient (CC) during HAL and CC during NoHAL in the next session (top left), CC during NoHAL and CC during HAL in the next session (bottom left), global efficiency (GE) during HAL and GE during NoHAL in the next session (top right), and GE during NoHAL and GE during HAL in the next session (bottom right). The scatter plot is plotted and regressed for the averages on the lower frequency ranges (blue, 2–22 Hz) and higher frequency ranges (green, 22–44 Hz).

Muscle network analysis showed the PSD residing mostly below 40 Hz (Figure 6A). IMCOH varied through the sessions for NoHAL and HAL (Figure 6B). CC and GE showed a statistically significant correlation in linear regression between CC during HAL and CC during NoHAL of the next session (*p* = 0.0079178 < 0.01, *R*^2^ = 0.785; Figure 6C, top left), as well as in regression between GE during HAL and GE during NoHAL of the next session (*p* = 0.0088224 < 0.01, *R*^2^ = 0.776; Figure 6C, top right). Conversely, no statistical significance was observed in the regression between CC during NoHAL and CC during HAL in the next session (*p* = 0.68, *R*^2^ = 0.039). It was also the case for GE (*p* = 0.66, *R*^2^ = 0.041; Figure 6C, bottom row).

## Discussion

In order to evaluate the effect of gait training using robot suit HAL, gait and lower limb muscle activity were recorded through HAL sessions and analyzed in a thoracic myelopathy patient who experienced a severe sensory motor paralysis and gait impairment before and after spine surgery. After the HAL sessions, the patient improved gait, with greater range of joint motion, faster walking speed, and greater step length (Figure 3). After transferring to another hospital, the patient was able to walk independently using a cane 2 months after HAL and achieved independent walking without a cane 11 months after HAL.

Disturbed gait of cervical myelopathy patients has been reported in the literature, characterized by a slower walking speed, shorter step length, longer step time, reduced range of joint motions (62–64), and altered timing and duration of muscle activities (65). After surgery for cervical decompression, improvement is observed in walking speed, step time, step length, and joint motions (66–68) and in knee kinetics (69). While the existing studies dealt with cervical myelopathy patients who could ambulate autonomously before and after surgery, in this study, we dealt with a thoracic myelopathy patient who had difficulty walking before and after surgery. When the patient became able to take a few steps, walking speed was slow, step length was short, and the range of joint motion was small (Figures 2, 3). These variables were improved after finishing the HAL training (Figures 2, 3).

In the second HAL session, when the patient started to walk using the walking device, he needed a large amount of weight support and could barely move his legs. The step length was very small without HAL. The activity of the measured muscles was also minor without HAL (Figure 5A, top row). However, using HAL, the step length was greater (Figure 4, middle) and the extensor muscles (quadriceps and gluteus maximus) were activated more during the stance phase than without HAL (Figure 5A, second row and Figure 5B, left). The activation ratio of HAL against NoHAL of these muscles was >1 (Figure 5C). This is contrary to the idea that muscle activations might reduce with robotic assistance (70). We consider that it is because, due to HAL's assistance, the muscle activities were made functionally effective in the actual performance of gait generation and that it generated an activity-based sensory feedback to the neural system, possibly leading to plasticity in the sensorimotor activity of the spinal locomotor network (35) and supraspinal networks related to gait control (36). Gait parameters of walking speed, step length, and percentage of stance duration showed greater values in HAL than in NoHAL (Figure 4). The greater limb motions could have led to sensory signal-driven activation of the spinal cord by the proprioceptive feedback (71).

Ivanenko et al. (72) showed changes in the amplitude of EMG activation of the lower limb muscles along with increased body weight support in healthy participants, demonstrating the importance of sensory information of the plantar pressure on the sole of the foot for the generation of normal muscular activity. Coming back to our patient, the patient's weight-bearing was very weak without HAL, so the weight was mostly supported by the unweighing device. Contrarily with HAL, with the assistance of HAL which generates joint torques by amplifying the sensed muscle activity, the patient was able to better bear weight on his feet. HAL assists the patient's weight-bearing only when the patient's extensor muscles are active, and this assures consistent signaling of sensorimotor interactions in the patient's central nervous system. Passive weight support devices, for example long-leg brace (LLB), which completely locks the knee joint, can support the weight-bearing of patients with weak knee joint control. However, in this case, the patient does not need to activate the knee extensor muscles since the rigid frame of the LLB supports the weight irrespective of the muscle activity. Shimizu et al. (73) showed the effectiveness of HAL over LLB for the activation of lower limb muscles in spinal cord injury patients. The contribution of the step signal-dependent afferent information to the neural control of locomotion by the spinal cord is considered, based on observation of a loading and velocity-dependent increase of EMG activity in spinal cord injury patients with reduced supraspinal input (74).

In the following sessions, the iEMG ratios were slightly <1 in the fourth and fifth sessions (Figure 5C), but the gait assisted by HAL showed greater step length (fourth session; Figure 4, middle) or percentage of swing duration (fifth session; Figure 4, right), which are a necessary characteristic of larger gait, and it continued through the rest of the sessions. In the later sessions, the iEMG ratio was reduced in the quadriceps and increased in the gluteus maximus using HAL (Figures 5A–C). It was represented by gait, by its faster speed and greater step length and percentage of swing duration walking with HAL (Figure 4). The percentage of swing duration was comparative between the initial session without HAL and the later sessions with HAL. We consider that the increased activity of the hip extensor contributed to stance stability for better swing motion of a contralateral leg as well as to faster propulsion. The reduced activity of the knee extensor contributed to a smoother landing and shock absorption by double knee action. These were observed in the POST gait without HAL (Figure 2), having greater joint range of movement and closer to normal angular profiles, including a smooth double knee action during the initial to mid-stance phase. The activation ratio of HAL against NoHAL was closer to 1 in the later sessions in comparison to the initial session (Figure 5C). This is because the weight-bearing without HAL mentioned above was not an issue in the later sessions. Adjustment of muscle activity for achieving larger steps in the later sessions did not cause such a drastic change in the amplitude of muscle activity.

Muscle synergy analysis showed significantly smaller VAF in HAL when the number of synergies were restricted to one or two (Figure 5D, right bottom), suggesting that a greater number of basic synergy patterns was incorporated during walking with HAL. Reduced number of muscle synergies is known in locomotion of incomplete spinal cord injury patients (51) and of stroke patients (75). Lack of muscle synergies corresponding to posture control is known in spinalized cats (76). The neural implementation of muscle synergies is reported as being housed in the spinal cord (44, 77), and the brain is also considered as forming a spatiotemporal pattern of synergies (45). Considering these evidences, increased synergies during walking with HAL suggest, first, that the spinal control of gait is altered with HAL and, second, the myelopathic spinal cord of the patient is activated with HAL to deal with more degrees of control in view of the simplified synergies of neurological patients (47, 51, 75, 78), together with the adapted activity of the supraspinal networks utilizing the increased synergy of the spinal cord to control gait (36, 46).

Muscle network analysis showed a statistically significant correlation of CC and GE during HAL to those during NoHAL in the next session (Figure 6C, top row). But a similar analysis on the correlation of CC and GE during NoHAL and those during HAL in the next session did not show statistical significance (Figure 6C, bottom row). Considering that CC and GE respectively indicates the extent of functional segregation and integration of a network (61), the result suggests that the muscle network property is modulated during HAL, and the modulated gross structure is maintained until the next session. Naro et al. (55) showed that CC and GE during gait of patients with a type of muscular dystrophy are different from those of healthy people and discussed that their gait problem not only depends on the deterioration of the muscles but also on the deterioration of the signaling system of the muscle network utilized by the central nervous system. The modulation of CC and GE with HAL also suggests modulated control of muscles by the central nervous system during walking with HAL. Recent studies on the cortical control of muscle synergies discuss that beta-band frequency (13–30 Hz) and piper rhythm frequency (around 40 Hz) are used for communication between the cortical networks and the spinal cord for the purpose of formation, control, and maintenance of muscle synergies (79, 80). Our muscle network analysis used a frequency band of 22–44 Hz as the higher part of frequency band based on the PSD of each muscle. The modulation of the muscle network took place in this frequency band, suggesting a relationship between the modulation of muscle synergy and muscle network during walking with HAL, though further detailed investigation is necessary to clarify this relationship.

There are some reports on neural activity changes by HAL in recent literature: brain activity changes in the primary motor cortex of subacute stroke patients immediately after using HAL (81), cortical excitability changes in the primary somatosensory cortex of spinal cord injury patients after 3 months of training (16), muscle synergy changes in the lower limbs of stroke patients (50), segmental coordination changes in in the lower limbs of myelopathy patients after surgery for thoracic OPLL (11), and activation in some muscles of chronic spinal cord injury patients during and after HAL training (17).

Israel et al. (70) observed that, during walking with a robot which autonomously assists limb motion (hence, the legs were passively moved), activation of leg muscles was significantly reduced. They discussed that the reduction is not effective for activity-dependent plasticity of the spinal and supraspinal locomotor circuitry (34). In this study, we reported an increased muscular activity at the initial sessions and commitment of more synergy patterns during walking with HAL throughout the sessions. Instantaneous change of muscle activation and synergies by using HAL in each session suggested that the changes are in the neural control rather than musculature. Indeed, the muscle signaling network was modulated during walking with HAL and sustained until the next session, suggesting that a greater extent of plasticity may be taking part during HAL walking. It was considered that HAL's assistance based on bioelectric signals might have helped induce modulation in the control of the muscles by the central nervous system, non-invasively. Studies using invasive methods showed the importance of feeding proprioceptive sensory signal to the spinal cord for recovery of supraspinal control and locomotion (82, 83). Activity-based sensorimotor augmentation by HAL can be a non-invasive method to effectively alter central control of gait.

A limitation of the study is that, as a case report, the data are from only one subject. Generalization of the results is not reasonable, considering broad variations of gait impairment. As shown by our results, muscular activity modulation by the robot varies through the course of recovery. Future studies may include examination of muscular activity modulation in multiple subjects of various levels of gait impairment and recovery.

Another limitation of the study may be that mechanical parameters such as HAL's assistance level (gain parameters of the equations in section Hybrid Assistive Limb) were not recorded. On the other hand, from the view point of neuro-rehabilitation, neuromechanical observations from physiological data rather directly evaluate changes in neural control of gait. Advances in the analysis of muscle activity during assisted movements (84–86) may help further research in this direction.

In conclusion, the muscular activity modulation by HAL observed in a patient with gait impairment due to severe myelopathy suggested that HAL may be effective in modulating central control of gait of the patient by its activity-based sensorimotor augmentation. The relationship between the altered gait control during walking with HAL and achievement of gait improvement requires further investigation with more cases.

## Data Availability Statement

The datasets generated for this study are available on reasonable request to the corresponding author.

## Ethics Statement

This study was approved by the University of Tsukuba Hospital Ethics Committee (approval number H26-22) and implemented according to the ethical principles of the Declaration of Helsinki and the University Guidelines for Clinical Trials. Before inclusion in the study, the patient received explanation of the research contents and data usage, for which written informed consent was obtained. At the time of submission of the manuscript, the patient was shown the manuscript and the images, and received explanation according to the Frontiers consent form, which meets the ICMJE guidelines. The patient gave a written agreement to a Japanese version of the Frontiers consent form (translation done by the authors).

## Author Contributions

HK collected, analyzed, and interpreted the data, and wrote and drafted the article. SK administered HAL treatment and collected the clinical scores. TA and HN diagnosed and operated the patient and prescribed HAL treatment. TA, KM, YSh, and MY reviewed and edited the article. KM, MK, YSh, and YH provided important comments on the planning and implementation of HAL treatment. YSa originally developed the robot suit HAL and conceived the idea of HAL treatment. KS provided ideas on clinical gait analysis. MY organized the study. All authors made critical revisions of the manuscript and approved the final version.

### Conflict of Interest

YSa is the C.E.O., shareholder, and director of CYBERDYNE Inc., which produces the robot suit HAL. CYBERDYNE was not involved in the study design, data collection, analysis, writing, or submission of this article. The remaining authors declare that the research was conducted in the absence of any commercial or financial relationships that could be construed as a potential conflict of interest.
