# Supplementary material for: Muscular Activity Modulation During Post-operative Walking With Hybrid Assistive Limb (HAL) in a Patient With Thoracic Myelopathy Due to Ossification of Posterior Longitudinal Ligament: A Case Report
Source: Front Neurol. 2020 Mar 31;11:102. doi: 10.3389/fneur.2020.00102 (PMC7136555; doi:10.3389/fneur.2020.00102)
Supplement: Supplementary file 1 [file Data_Sheet_1.PDF]

Supplementary Material

1 Supplementary Figures

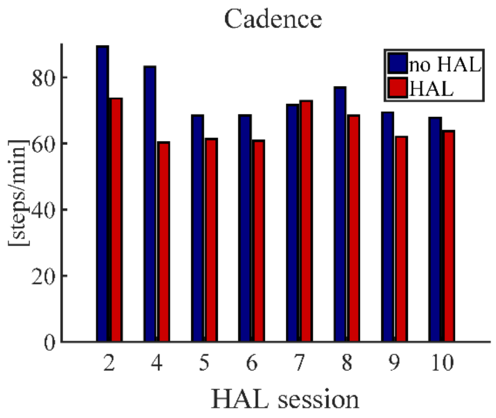

Supplementary Figure 1. Cadence during walking with HAL (HAL) and without HAL (NoHAL), in each of the HAL sessions.
